# Effect of Hydrothermal Treatment with Distilled Water on Titanium Alloy for Epithelial Cellular Attachment

**DOI:** 10.3390/ma12172748

**Published:** 2019-08-27

**Authors:** Yasushige Sakamoto, Yasunori Ayukawa, Akihiro Furuhashi, Michimasa Kamo, Junji Ikeda, Ikiru Atsuta, Takuya Haraguchi, Kiyoshi Koyano

**Affiliations:** 1 Section of Implant and Rehabilitative Dentistry, Division of Oral Rehabilitation, Faculty of Dental Science, Kyushu University, Fukuoka 812-8582, Japan; 2 Medical Division, KYOCERA Corporation, Shiga 520-2362, Japan; 3 Division of Advanced Dental Devices and Therapeutics, Faculty of Dental Science, Kyushu University, Fukuoka 812-8582, Japan

**Keywords:** hydrothermal treatment, cell adhesion, Ti-6Al-4V, epithelial GE1 cells, laminin-332

## Abstract

The enhancement of oral epithelial adhesion to the trans-mucosal material of dental implants may improve their long-term stability. The aim of this study is to investigate whether hydrothermal treatment with distilled water (HT-DW) applied to a Ti-6Al-4V (Ti64) alloy could improve epithelial cellular attachment. We hypothesized that this treatment would enhance the adsorption of proteins and the adhesion of gingival epithelial GE1 cells. This treatment changed the surface crystal structure into an anatase type of titanium oxide without an apparent change of surface roughness or topography. Nitrogen was not detected on the HT-DW-treated Ti64, which indicates decontamination. HT-DW-treated Ti64 exhibited a hydrophilic surface with a less than 10° angle of water contact. Adsorption of laminin-332 to the HT-DW-treated Ti64 was significantly greater than that of the untreated Ti64 plates (64). The number of GE1 cells on the HT-DW-treated Ti64 at 1 and 3 days was significantly lower than that on 64; however, cell adhesion strength on HT-DW was greater, with a higher expression of integrin β4, compared with 64. This indicates that the HT-DW treatment of Ti64 improves the integration of GE1 cells, which might facilitate the development of a soft tissue barrier around the implant.

## 1. Introduction

Dental implants are placed in the jaw bone and penetrate the oral mucosa. They function within, and are exposed to, the oral cavity. Peri-implant soft tissue is known to have a “biologic width” having some features in common with the epithelial and connective tissue of natural teeth [1]. The establishment of a firm, functional soft tissue barrier is believed to be important in protecting the tissue–implant interface from bacterial invasion [2]. Peri-implant soft tissue is characterized by a scar-like structure [3]. This may be the reason for peri-implant epithelial sealing being weaker against external stimuli than that of natural teeth [4].

Previous studies have shown that the topography of the implant surface affects cell adhesion [5,6]. A rough surface that facilitates the adhesion and differentiation of osteoblasts helps in promoting osseointegration [7,8]. However, a rough surface has inferior adhesion of epithelial tissue [9,10], easily accumulates a biofilm [11], and is subject to the aggressive progress of peri-implantitis when compared to a smooth surface [12]. Thus, smooth surfaces are generally used for the trans-mucosal portion of the implant, which has direct contact with soft tissue.

As titanium (Ti) has moderate mechanical properties and superior biocompatibility, it has been used as an implant material for many years [13]. However, as the use of dental implants has increased, severe cases, such as patients with parafunction or with a limited dimension of their alveolar ridge in buccolingual and mesiodistal dimensions, require greater mechanical strength of their implants. Because fracture of the implant body has been reported [14], a Ti alloy with greater mechanical strength has been used as an implant material. In addition, Ti alloy is also utilized as an abutment material because abutment carries the possibility of damage by external factors, such as tooth brushing and scaling. Among Ti alloys, Ti-6Al-4V (Ti64), which contains 6% Al and 4% V, has more tensile strength compared to pure Ti [15], which is most commonly used as an implant or abutment material. However, the biological evaluation of Ti64, especially for soft tissue, has not been well reported.

One of the important roles of the abutment is to protect the dental implant from bacteria in the oral cavity. It is reported that the size and/or shape of the abutment affects the soft tissue around the implant [14]. Recently, customized abutment has been widely used for its adjustability to the soft tissue contour. In this context, if the surface treatment can be easily applied to customized and/or ready-made abutments, the clinical benefit might be significant.

Among the many surface modifications of metallic biomaterials, it has been reported that the hydrothermal treatment of Ti alloys increases surface hydrophilicity and improves osteoconductivity [15]. This treatment can be applied to materials easily. In addition, it requires an oven, pressure pot, and water, and all of these can be obtained inexpensively.

In the present study, we hypothesize that the hydrothermal treatment of Ti64 may promote the adhesion of epithelial cells. The aim of this study is to determine the efficacy of the hydrothermal treatment of Ti64 for epithelial attachment.

## 2. Materials and Methods

### 2.1. Treatment of Titanium Alloy Plates

Machined titanium alloy (Ti64 ELI: ASTM F136) and commercially pure titanium (TiCP ASTM F67 Grade2) plates with flat surfaces, 15 mm in diameter and 1 mm in thickness, were polished using SiC emery paper (Silicon Carbide Grinding Paper, Grit 2000, Struers ApS, Ballerup, Denmark). They were then washed in 100% acetone, distilled water, and 99.5% ethanol in an ultrasonic bath before γ-ray sterilization. The hydrothermal treatment with distilled water (DW) group was treated using 15 mL of DW on Ti64 in a hydrothermal unit (HU-50, SAN-AI Kagaku, Nagoya, Japan) at 200 °C for 24 h (64HT) [16,17]. The control group received no treatment on Ti64 (64). The commercially pure group received no treatment on TiCP (CP). After treatment, each specimen was washed with DW for 10 s and stored in a vacuum desiccator to prevent surface contamination until being washed and sterilized, as described above.

### 2.2. Surface Characterization

The surface morphology of the Ti64 specimens was examined using a scanning electron microscope (S-3400N, Hitachi High-Technologies, Tokyo, Japan) at an accelerating voltage of 15 kV. The surface roughness (Ra) and maximum roughness height (Rt) were measured in a surface roughness measurement microscope (SV-3100, Mitutoyo, Kanagawa, Japan). For Ra and Rt, five randomly chosen spots per sample, two samples per group, were measured (n = 10 for each group). The crystal structure of surface titanium oxide was analyzed at a wavelength of 514.77 nm using a laser Raman microspectrograph (LabRAM; HR-800, Horiba, Kyoto, Japan).

Nitrogen 1s, which indicates the surface contamination of specimens, was analyzed by X-ray photoemission spectrometry (K-alpha, Thermo Fisher Scientific, East Grinstead, UK). All binding energies were referenced to the carbon 1s component set to 285.0 eV.

The hydrophilic status of the Ti surfaces was measured by the contact angle of a 1 µL droplet of distilled water in place for 60 s by a contact angle meter (Drop Master 500FK, Kyowa Interface Science, Niiza, Japan) (n = 6 for each group).

### 2.3. Protein Adsorption Assay

Recombinant Human laminin-332 (Ln; ReproCELL, Yokohama, Japan) was used as the model protein. All procedures for the adsorption assay were performed at room temperature (RT). Ti64 plates (64 and 64HT) were immersed in 0.5 µg/mL diluted Ln in phosphate-buffered saline (PBS) for 1 h. After rinsing off the unbound protein with PBS, the plates were treated with 10% normal goat serum (Nichirei Bioscience, Tokyo, Japan) in PBS for 10 min to prevent nonspecific adsorption of antibodies. The plates were then incubated with 4 µg/mL diluted mouse anti-human Ln monoclonal antibody (P3H9-2, Santa Cruz Biotechnology, Dallas, TX, USA) in PBS for 1 h. After rinsing off the unbound antibodies with PBS, the plates were treated with 10 µg/mL diluted fluorescein isothiocyanate (FITC)-conjugated goat anti-mouse IgG secondary antibody (Invitrogen, Carlsbad, CA, USA) in PBS for 30 min. The plates were then rinsed with PBS, and the fluorescent intensity was measured at an excitation wavelength of 485 nm and an emission wavelength of 535 nm using a microplate reader (Infinite F200PRO; Tecan, Salzburg, Austria) (n = 6 for each group).

### 2.4. Cell Culture

**Figure 1:**
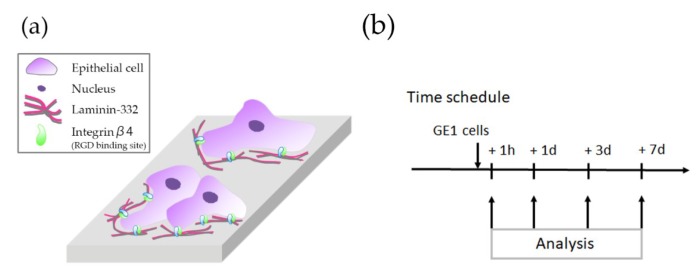
(**a**) Schematic diagram of the epithelial cells. (**b**) Experimental protocol for the in vitro experiments.

The GE1 (RIKEN Cell Bank, Tsukuba, Japan) mouse-derived gingival epithelial cell line was used (Figure 1a). GE1 cells were cultured in basal serum-free medium (SFM-101, Nissui, Tokyo, Japan) containing 1% fetal bovine serum (Biowest, Nuaillé, France) and 10 ng/mL mouse epidermal growth factor (Corning, New York, NY, USA) in a humidified atmosphere with 5% CO_2_ at 33 °C. Cells were seeded within a 1 mL volume onto each Ti plate, at a density of 5 × 10^4^ cells per well in a 24 well culture plate (Multiwell 24 well, Corning).

### 2.5. Cell Number Measurement

The number of attached cells was measured using a cell count kit (Cell Count Reagent SF, Nacalai Tesque, Kyoto, Japan). The cell number was counted at 1 h, 1 day, 3 days, and 7 days following seeding (Figure 1b). The culture medium was removed and replaced with 1.1 mL of culture medium containing 100 µL of Cell Count Reagent SF. After incubation at 33 °C for 2 h, the solution was removed, and the reaction was stopped with 10 µL of 0.1 M hydrogen chloride. The absorbance was measured at 450 nm using a spectrophotometer (NJ-2300, Biotech, Tokyo, Japan) (n = 6 for each group). 

### 2.6. Adhesion Assays

The adhesion strength of GE1 cells was expressed by the cell adhesion ratio, as reported previously [18,19]. Non-adherent, weakly-attached, and dead cells were removed by shaking (six times for 10 min at 160 rpm) using a rotary shaker (NX-20, Nissin, Tokyo, Japan) immediately following the initial cell count after 1 day of culture. The remaining adherent cells were measured using the Cell Count Reagent SF and the percentage to the initial cell count was defined as the cell adhesion ratio (n = 6 for each group).

### 2.7. Immunofluorescent Staining for Adhesion Proteins

GE1 cells on the experimental plates, after 1 day of culture, were fixed with 4% formaldehyde (Merck, Darmstadt, Germany) for 10 min, blocked with 1% bovine serum albumin (BSA; Bovine Serum Albumin Fraction V, Roche Diagnostics, Basel, Switzerland) for 30 min at RT, and then incubated overnight at 4 °C with a 1:200 dilution in BSA of goat anti-rat integrin β4 (In-β4) polyclonal antibody (C-20, Santa Cruz Biotechnology). After washing with PBS (5 min × 3 times), the cells were labeled for 2 h at RT with a 1:200 dilution in BSA of FITC-conjugated anti-goat IgG secondary antibody (Invitrogen). Actin filaments were stained for 1 h at RT with a 1:100 dilution in BSA of tetramethylrhodamine isothiocyanate (TRITC)-conjugated phalloidin (Sigma-Aldrich, St. Louis, MO, USA). The cells were then mounted with anti-fade reagent containing 4′6-diamidino-2-phenylindole (DAPI; VECTASHIELD, Vector Laboratories, Burlingame, CA, USA) for nuclear staining. The stained cells were observed under a fluorescence microscope (BZ-9000, Keyence, Osaka, Japan).

### 2.8. Statistical Analysis

Data are expressed as the mean ± standard deviation (SD). Student’s *t*-test or one-way analysis of variance (ANOVA) with Tukey’s method (for multiple comparison) was performed. Values of *p* < 0.05 were considered statistically significant.

## 3. Results

### 3.1. Characterization of the Materials

**Figure 2:**
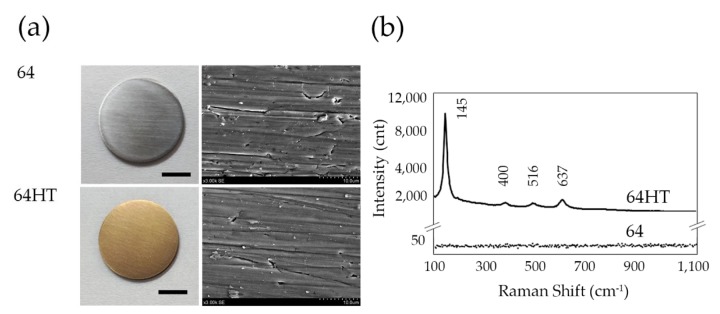

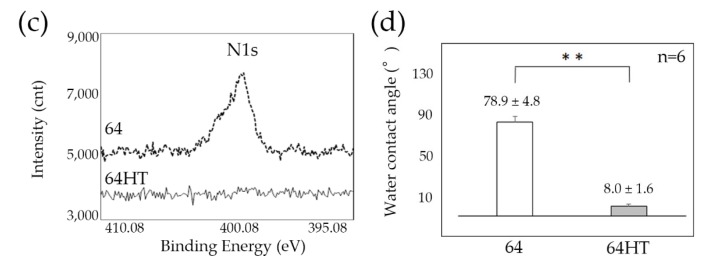
(**a**) Experimental plates for the control group (64) and the hydrothermal treatment group (64HT) (bar = 5 mm) (left). Scanning electron microscope images of titanium surfaces (bar = 10 µm) (right). (**b**) Raman spectrum of 64 and 64HT. (**c**) Nitrogen peak fitting of the XPS narrow scan spectra of 64 and 64HT. (**d**) Water contact angle for the evaluation of 64 and 64HT surface wettability. 64: untreated Ti-6Al-4V; 64HT: hydrothermally treated Ti-6Al-4V in distilled water at 200 °C for 24 h. Data are shown as the mean ± SD. Statistical analysis was by *t*-test (** *p* < 0.01).

There were no statistically significant differences in the Ra and Rt values between 64 and 64HT (Table 1). The 64HT samples exhibited a gold color (Figure 2a). SEM observation at magnification of 3000 times showed no clear change after the 64HT treatment (Figure 2a), and the grooves of mechanical polishing could be seen. In surface crystal structure analysis, 64 was similar to an amorphous peak which appears non-crystalline, and 64HT was similar to an anatase-type titanium oxide peak (Figure 2b). N1s peak in the XPS spectra of the 64 was observed around 400 eV on the surface, but was not observed on 64HT (Figure 2c). The water contact angle for the evaluation of surface wettability on the 64HT was significantly lower than that on the 64 (Figure 2d). 

### 3.2. Amount of Adsorbed Ln

**Figure 3:**
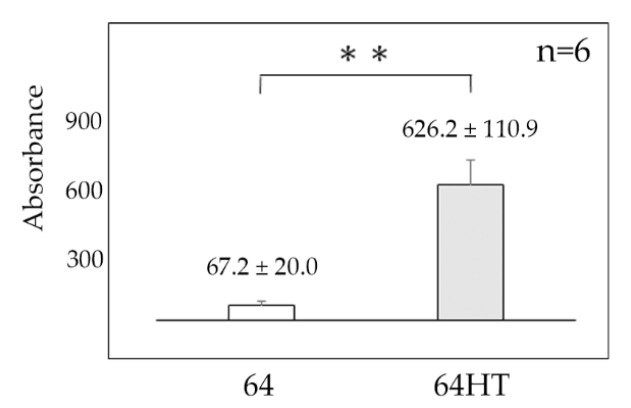
The adsorbed amount of laminin-332 on the surface of 64 and 64HT plates. Data are shown as the mean ± SD. Statistical analysis was by *t*-test (** *p* < 0.01).

Figure 3 shows the amount of Ln adsorbed onto control and 64HT plates.

In the 64HT samples, the amount of adsorbed Ln was significantly greater than that in the 64 samples.

### 3.3. GE1 Initial Attachment and Proliferation

**Figure 4:**
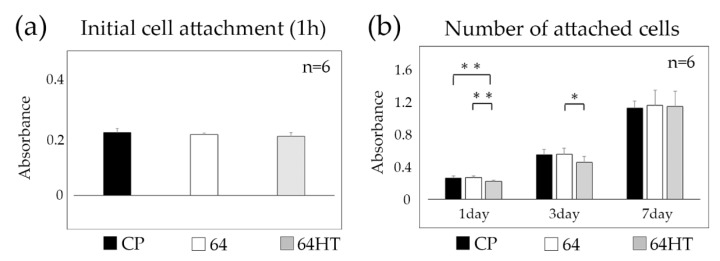
(**a**) Initial attachment of GE1 cells was analyzed 1 h following seeding. (**b**) The number of GE1 cells was analyzed after 1, 3, and 7 days of incubation. Data are shown as the mean ± SD. Statistical analysis was by ANOVA with Tukey’s test (* *p* < 0.05, ** *p* < 0.01).

No statistically significant differences were found in the numbers of initially attached GE1 cells among all groups (Figure 4a). The number of cells, which indicates the proliferation on the substrata, was chronologically increased in all groups. The numbers of cells on 64 on the substrate were significantly greater than those on 64HT after 1 day. After 3 days, the number of cells on 64 was significantly greater than that on 64HT, whereas no significant difference was observed at day 7 (Figure 4b).

### 3.4. Adhesion of GE1 Cells

**Figure 5:**
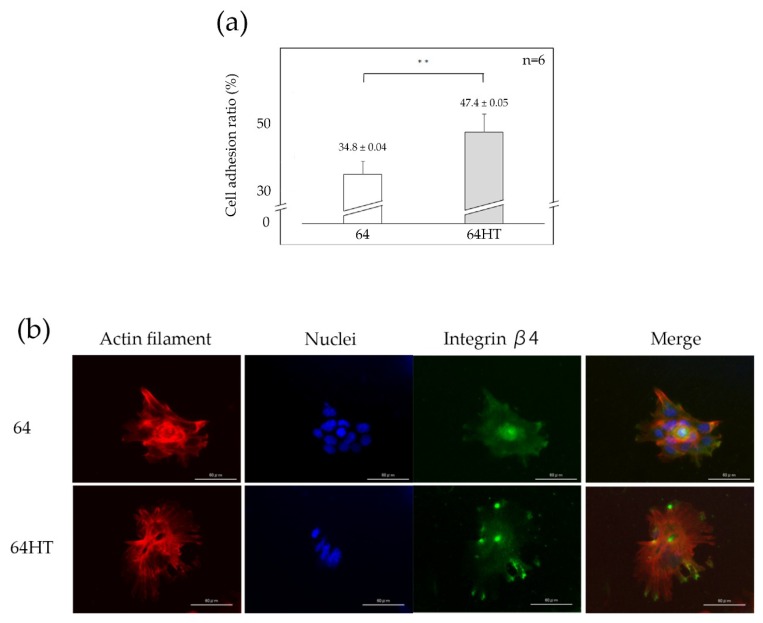
(**a**) Adhesion assay of GE1 cells. Data are shown as the mean ± SD. Statistical analysis was by *t*-test (** *p* < 0.01). (**b**) Immunofluorescence staining of GE1. Actin filaments (red), nuclei (blue), and integrin β4 (green) are shown (bar = 60 µm).

We provided external vibration to plates and detached weakly adhered or dead cells, then remaining adherent cells on the plates were counted and the percentage to the initial cell count was defined as the cell adhesion ratio. The cell adhesion ratio was greater in the 64HT group than in the 64 group (Figure 5a). 

### 3.5. Expression of In-β4, Nucleus, and Actin Filaments of the GE1 Cells

GE1 cells were cultured on the 64 or 64HT plates for 1 day and observed with a fluorescence microscope (Figure 5b). Actin filaments at the intracellular margin of the cells in both groups were observed. Additionally, a stronger signal of In-β4 at the periphery of the cells in the 64HT group was observed (Figure 5b). 

## 4. Discussion

Materials used for dental implants need to have not only the mechanical ability to support an occlusal force but also the chemical properties of biocompatibility and corrosion resistance [20]. A pure titanium surface will form an oxidized layer immediately following exposure to air, which is quite stable [21]. It has been shown that multiple types of cells can proliferate and adhere to pure titanium [22,23,24,25]. Therefore, pure titanium, with its excellent biocompatibility and corrosion resistance, has been widely used as a biocompatible material for more than half a century [13].

Commercially, titanium has been divided into four classes, ranging from pure titanium to various degrees of titanium alloys. Greater mechanical strength has been reported, with an increase in the amount of other elements compared to that of titanium. Since Ti64, which includes 6% Al and 4% V, shows greater tensile strength than pure Ti [26], it has clear advantages in terms of its mechanical properties [27]. At the same time, the biological compatibility of aluminum and vanadium must be carefully evaluated.

In some previous studies, it was reported that there is no significant difference of osteoblast behavior on cell adhesion or bone to implant contact between pure Ti and Ti64 [28,29]. We found no significant difference in the number of initial cell attachments and cell proliferation between CP titanium and Ti64 (Figure 4a,b). For this reason, Ti64 might be as good as CP titanium relative to their biocompatibility.

It has been reported that the resistance of peri-implant soft tissue to exogenous factors is weaker than that of periodontal tissue. It has also been suggested that improved attachment between epithelial cells and the titanium surface can reduce the risk of infections [14,30]. We therefore investigated the effect of the hydrothermal treatment of Ti64 on epithelial cell behavior.

The rough surface of dental implants was reported to inhibit the proliferation and attachment of gingival epithelial cells or fibroblasts [6,9,31], facilitate biofilm or bacterial adhesion [32], and cause a greater occurrence of peri-implantitis [12]. Moreover, the effect of surface topography on bacterial attachment was related to the irregularities of the size of the roughened surface. A concavity distance of 3 µm accumulates more biofilm, whereas less accumulation has been observed with a concavity under 0.4 µm [33]. In our study, surface roughness was not increased after 64HT treatment, implying favorable characteristics with regard to biofilm accumulation.

The oxide layer of the titanium surface can be divided into an amorphous type, which appears non-crystalline and anatase, and a rutile type that varies in crystalline structure [34]. The anatase type forms a hydroxyl group on the surface, which makes the surface hydrophilic [35]. It has been reported that the adsorption of adhesion proteins increases on hydrophilic surfaces through the attachment of the hydroxyl group on the titanium surface [36,37]. In the present study, the hydrothermal treatment of titanium in water was employed. This treatment modified the surface oxide layer (Figure 2b), removing depositions on the surface (Figure 2c), and as a result, the surface hydrophilicity was improved (Figure 2d). Thus, this treatment ameliorated the epithelium adhesion onto titanium via an easy and low-cost procedure. When cells adhere to the substrate, integrin on the cell membrane adheres through a matrix protein such as laminin [38]. Our data showed increased adsorption of Ln on the HT-DW-treated Ti64 surface and enhanced expression of In-β4 at the periphery of GE1 cells, which indicated stronger adhesion of the cells.

Peri-implant soft tissue is known to have some features in common with the epithelial and connective tissue of natural teeth, and the peri-implant epithelium (PIE) is known to be similar to the junctional epithelium (JE) [1]. In cells of the JE, the binding of integrin α6β4 (In-α6β4), which is a transmembrane protein to Ln localized within the basement membrane, plays a major role in the organization of hemidesmosomes and epidermal cell-basal lamina adhesion [39]. It has been reported that a basal membrane-like structure is present at the PIE around untreated machined pure titanium, but there is no abundance of Ln and expression of In-α6β4 [40].

The number of cells on the HT-DW-treated surface was significantly lower after 1 day and 3 days of culture (Figure 4b). At day 7, since the cells on all Ti plates became confluent, no significant differences in cell numbers were observed among all groups. However, the strength of their attachment was significantly greater on the HT-DW-treated surface (Figure 5a), and the expression of In-β4 was stronger than that in the control group (Figure 5b). It has been reported that integrin not only is involved in the regulation of cell–cell and cell–substrate contact, but also has an influence on cellular function by intracellular signal transduction, such as cellular extension, movement, differentiation, and proliferation [41]. The results of our study led us to presume that cell proliferation on the HT-DW-treated surface at 1 day and 3 days was inhibited by the improved cell–substrate adhesion at the initial stage. Thus, it could provide better epithelial adhesion and may protect against the various stimuli encountered in clinical applications.

The limitation of this study is that we tested only one processing time and temperature. In the pilot experiment of our previous study using bone tissue, we tried several different temperatures and processing times [17]. It was found that temperatures lower than 200 °C and processing times shorter than 24 h resulted in inferior bone tissue attachment to titanium. Both temperatures higher than 200 °C and processing times longer than 24 h are inappropriate as the operating environment for the pressure pot used here and in our previous studies. Thus, in the present study we selected the temperature and processing time without any pilot experiment. For simple and cost-effective processing, treatment with lower temperatures and shorter processing times is valuable. We would like to try several different processing conditions in future study. Another limitation is that we used plates with machined surfaces, as we would like to eliminate factors other than hydrothermal treatment to assess epithelial cell attachment. However, some recent abutments have nano-textured or grooved surfaces. The further study of Ti hydrothermal treatment using textured surfaces is expected.

## 5. Conclusions

The results of this study indicate that the hydrothermal treatment of Ti64 can impart hydrophilicity with minimal change to surface topography and improve the amount of Ln adsorption. Subsequently, this treatment enhanced the expression of In-β4 and the undetachability of epithelial cells from Ti64. In addition, this treatment can be easily applied to implant materials, even in clinical situations, with inexpensive facilities. This treatment has the potential to contribute to better epithelial tissue sealing around a dental implant, and may contribute to the maintenance of the health of soft and hard tissue around the implant. 

## Figures and Tables

**Table 1:** Surface roughness data of the Ti-6Al-4V samples. Data represent mean ± SD (n = 10).

Material	Ra (µm)	Rt (µm)
64	0.070 ± 0.008	1.03 ± 0.23
64HT	0.072 ± 0.010	0.95 ± 0.24
